# Assessing the Relationship between In-Water Kinetic Asymmetries and Performance in Swimming

**DOI:** 10.5114/jhk/211343

**Published:** 2025-11-20

**Authors:** Knihs Débora A, Chris Bishop, Luiz G. A. Guglielmo, Juliano Dal Pupo

**Affiliations:** 1 Biomechanics Laboratory, Centre of Sports, Federal University of Santa Catarina, Florianopolis, Brazil.; 2 Faculty of Science and Technology, London Sports Institute, Middlesex University, London, United Kingdom.

**Keywords:** force, impulse, swimmers, between-limb differences, biomechanics

## Abstract

The present study aimed to investigate the relationship among force, impulse and their asymmetries with swimming performance across different distances, and to explore whether the correlations were dependent on the presence of “real” asymmetries (i.e., higher than metric variability). Thirty-five male swimmers (age: 19.5 years ± 5.0, body mass: 75.4 ± 11.5 kg, body height: 181.7 ± 7.6 cm) performed a 15-s tethered-swimming test, in which peak force, mean force and impulse of each body side were measured. The absolute and relative asymmetries were subsequently obtained. The coefficient of variation was calculated and used to identify the presence of “real” asymmetries (i.e., asymmetries greater than the CV). The official times for 50-, 100-, and 200-m front-crawl swimming were obtained. Pearson’s r correlations showed that peak and mean force presented moderate to strong associations (r = −0.51 to −0.84; p ≤ 0.001 to 0.013) with performance at all distances. Impulse only presented moderate associations with 50-m front crawl performance (r = −0.50 to −0.62; p ≤ 0.001 to 0.013). When considering the whole sample, no associations were seen between asymmetries and swimming performance, but when considering only athletes presenting “real” asymmetries, weak to moderate correlations were found between peak force asymmetries and 50/100-m performance (r = 0.47 and 0.59; p = 0.016 and 0.002). In conclusion, force and impulse are related to swimming performance, and individual asymmetries should be monitored in swimmers, as they might be present and related to performance depending on the approach adopted.

## Introduction

The determinants of swimming performance have been frequently studied in recent years, especially in sprint swimming events, as any improvement may cause a meaningful difference (Barbosa et al., 2010; Price et al., 2024; Ruiz-Navarro et al., 2025). A variety of kinetic, kinematic and energetic factors are related to performance in swimming (Barbosa et al., 2010; Morais et al., 2023). Specifically, propulsion and drag are responsible for the swimmer’s ability to propel in the water (Santos et al., 2021). The amount of force applied in the water has been pointed as one of the key determinants of propulsion (Santos et al., 2021) and speed (Ruiz-Navarro et al., 2025), being associated with faster swimming times (dos Santos et al., 2017; Loturco et al., 2016; Rozi et al., 2018). Also, impulse (i.e., the force generated over time) is considered relevant in short-distance swimming events (Loturco et al., 2016), due to the importance of producing high amounts of force in short time frames. As a key sprint swimming performance indicator (dos Santos et al., 2013; Santos et al., 2021), assessing and monitoring in-water force and derived variables (such as impulse) in swimmers seems fundamental.

Inter-limb asymmetries (i.e., the difference in performance or function between body sides) are also suggested to influence swimming performance (dos Santos et al., 2013; Sanders et al., 2011). The rationale is that equivalent force application by both body sides might allow for higher total force output to be achieved, and also minimise the intra-cyclical velocity variation, helping maintain swimming speed (Santos et al., 2021). In addition, the similar “work” conducted between the body sides is also suggested to preserve body alignment in the water, minimising resistance drag (Sanders et al., 2011), which might further enhance performance. Thus, asymmetric force application between the body sides might be detrimental to swimming performance by affecting propulsion and resistance drag. However, despite this aforementioned narrative, the findings related to the presence of symmetry in swimming are controversial, with asymmetries in swimming speed reached by each upper limb being reported in sprint swimmers (Morais et al., 2020). Specifically, regarding the relationship between in-water asymmetries and performance, a small number of studies are seen, also with distinct results (Knihs et al., 2023). For example, dos Santos et al. (2013) showed that swimmers with better performance in the 200 m (fastest group vs. slower group) presented fewer asymmetries in peak and mean force during tethered swimming (*p* ≤ 0.05), while Morouço et al. (2015) found no differences (*p* > 0.05) in the 50-m performance between swimming athletes classified as symmetric (< 10%) or asymmetric (> 10%) in these variables. Also, asymmetries in force variables were not significantly associated with swimming velocity in any of the four swimming strokes (Bartolomeu et al., 2022). Thus, further research on the link between inter-limb differences and swimming performance is warranted.

Different methods have been proposed to evaluate the level of inter-limb asymmetries. Specific percentage cut-off values (e.g., 15%) are widely used in the literature, but have been criticised for being too general, disregarding the specificity of the asymmetries. When considering the asymmetries' task dependence, individuality and natural variability characteristics (Bishop et al., 2021), an approach that considers the specific metric variability and provides a more individual analysis can be advantageous. In this sense, the approach of contrasting the individual asymmetry’s value with the specific measurement variability’s value (e.g., coefficient of variation of the metric) has been suggested as a more reasonable alternative (Bishop et al., 2021; Exell et al., 2012; Phukan et al., 2021). In this approach, only asymmetries greater than the variability of the signal are considered “real”. A recent study used this approach to investigate the presence of inter-limb differences in swimmers and found that several of the tested athletes presented “real” in-water asymmetries (Knihs et al., 2024); however, not all participants showed these “real” asymmetries, and their presence also varied depending on the tested metric. That raises a question regarding the associations between asymmetries and performance, in which perhaps athletes with “unreal” asymmetries may distort the results (because of the “noise” created by the variability), altering the slope of the correlations. One alternative to this would be to analyse only athletes with asymmetries greater than the metric’s variability. For example, when considering only athletes with “real” asymmetries, the variability in the data is normally lower, which may allow correlations drawn with these data to be significant. To the author’s knowledge, that approach was not tested in swimming in previous literature and can bring relevant results, throwing light on future research and increasing the robustness of the analyses.

Furthermore, asymmetries can be calculated using a variety of methods (Bishop et al., 2016), including absolute differences (i.e., mean difference in raw scores) and relative differences (asymmetry % values). It is important to highlight that these different methods of analysis might provide different results due to the “noise” included in the calculation of each of them. Thus, investigating the association between swimming performance at different distances and: i) raw test scores, ii) absolute differences, and iii) inter-limb differences (asymmetry %), at the same time, may bring relevant results, being useful for swimming practitioners to determine where the strongest links exist (i.e., whether just in the raw metric or both raw metric and asymmetries), which, in turn, would help prioritize testing and monitoring practices.

Therefore, the present study aimed to i) analyse the relationship between force-related variables measured during tethered swimming (peak and mean force, impulse and their asymmetries) and swimming performance at different distances, and ii) investigate whether the correlations were dependent on the condition of the asymmetries (i.e., whether they were “real” asymmetries or not). The hypotheses were that i) the raw data would present correlations with swimming performance, but ii) the correlations between asymmetries and swimming performance would appear only when athletes with “real” asymmetries were analysed, due to the less variability.

## Methods

### Participants

Thirty-five male swimmers (age: 19.5 years ± 5.0, body mass: 75.4 ± 11.5 kg, body height: 181.7 ± 7.6 cm, arm span: 187.1 ± 9.4 cm) composed the sample. A G*Power posterior analysis showed that the number of participants was sufficient to reach a statistical power of 80 to 95% (depending on the number of participants for each analysis), with an alpha (α) of 0.05 and an effect size of 0.5. The competitive level of athletes varied among state (n = 6), national (n = 26), and international (n = 3). Swimmers trained 5.8 ± 0.5 days a week (4202 ± 1272 m per session) and had a training history of 8.2 ± 5.0 years. Of the 35 participants, 28 (80%) presented right-hand dominance, based on the answers to the Edinburgh Handedness Inventory (Espírito-Santo et al., 2017). Athletes reported no injuries in the past three months before testing. This research was conducted following the ethical standards of the Helsinki Declaration and was approved by the ethics committee of the Federal University of Santa Catarina, Florianopolis, Brazil (protocol code: 65671322.7.0000.0121; approval date: 02 March 2023). After being informed of the potential risks and benefits, participants and their parents/guardians (when < 18 years old) signed written informed consent to participate.

### Design and Procedures

In this cross-sectional study, we assessed the correlation of peak force, mean force, and impulse (both body sides), the respective absolute side-to-side differences of each metric, and their subsequent relative asymmetries measured during tethered swimming, with swimming performance at distances of 50-, 100- and 200-m front-crawl. The correlations were reached considering the complete sample and with only athletes that presented asymmetries higher than the coefficient of variation (i.e., meaningful asymmetries) to investigate further this approach.

On the first day, data collection was set in a biomechanics laboratory, where participants underwent anthropometric measurements. Following the International Society for the Advancement of Kinanthropometry protocol, body mass, body height, and the arm span were measured. A scale, a stadiometer, and a measuring tape fixed to the wall were used, respectively. The National Aquatic Confederation records were consulted to obtain the official best performances in the 50-, 100-, and 200-m front crawl in the period between the data collection and the closest date possible, with a limit of up to one year before. Most athletes (71.4%—50 m; 74.3%—100 m; 60.0%—200 m) had their best performance in a competition that took place three months before data collection. The performance of two athletes could not be obtained for the 200-m front crawl. On a different day, the tethered swimming test was conducted in an indoor 25-m swimming pool, with water temperature of 28°C. This test was selected because it allowed an in-water measurement of force applied for each limb, and provided a resultant inter-limb asymmetry metric as well. Although the tethered swimming test presents some differences in comparison to free swimming tests/events, such as: changes in the hand trajectory, the absence of water resistance, and stationary swimming (which does not allow to measure propulsion itself); it is an accessible, low-cost test, and widely used in previous swimming literature (dos Santos et al., 2013; Morouço et al., 2015; Santos et al., 2021).

Participants started by performing a warm-up composed, generally, of 300 m of front crawl swimming, 300 m of corrective swimming exercises, 200 m of kick exercises, and 200 m of velocity progressive exercise. That warm-up was used by them in competitions and varied slightly among athletes. After the warm-up, a 3-m inextensible rigid cable was tied between the swimmer’s waist and a load cell (AEPH Brazil, SP, Brazil – 200 kg), which was in turn, firmly tied to a swimming start block. Athletes swam tethered at low to moderate intensity until they familiarised themselves with the movement and were able to exert maximum performance, minimising the learning effect. A five-minute rest interval was provided between the familiarisation and the test to avoid fatigue. For the official test, each athlete performed three maximum attempts of 15-s front crawl tethered swimming, with intervals of 5 min between subsequent attempts. During the test, a synchronizer was manually triggered in every right-side stroke, emitting a pulse in the curves corresponding to the right side, allowing posterior identification of the sides for analysis. Breathing action and lower limb use were not controlled, being requested to athletes that they used the same patterns as in maximal events. All athletes chose to perform kicks, and most of them preferred not to breathe during the test.

### Data Analysis

The force curves were obtained during the test for each body side through the load cell, which was connected to a Miotool signal acquisition system (Miotec Equipamentos Biomédicos Ltda., Porto Alegre, Brazil – 2000 Hz). The force signals were treated and analysed by a mathematical routine implemented in the MATLAB software (Mathworks Inc., USA), being initially filtered with a 2^nd^ order Butterworth low-pass filter, with a cut-off frequency of 15 Hz (dos Santos, et al., 2013). In the force curves, each stroke was defined from the moment the force rose abruptly until it reached its lowest value. In each stroke, the following variables were analysed: a) peak force: considered the highest value of the resulting force (considering the angle of the cable in relation to the water level/horizontal plane); b) mean force: the mean values of the force curve; c) impulse: calculated by integrating the area of the force-time curve for each stroke (dos Santos et al., 2013). Three strokes (not necessarily consecutive) on each body side were manually selected in each attempt, and a mean was calculated to represent the mean’s attempt, for each body side. Then, the mean of the three attempts for each body side was considered for statistical analysis.

### Statistical Analysis

Excel and JASP software were used for the needed analysis. Firstly, the mean and standard deviation (SD) for all data were computed. The absolute difference between the body sides (maximum value – minimum value) and relative symmetry (%) for each metric were also calculated. The relative asymmetry (%) was determined through the following equation: percentage differences = 100 / (maximum values) * (minimum value) * −1 +100 below (Bishop et al., 2020).

To verify the presence of “real” asymmetries, initially, the individual coefficient of variation (CV = SD (attempts 1**−**3) / Mean (attempts 1–3) * 100) was calculated for each variable (absolute metric), on each body side, providing a measure of the metric variability. Then, the higher CV value (obtained on the right or the left side) was contrasted with the relative asymmetry (of the specific metric). The asymmetry was considered “real” only in athletes whose relative asymmetries were higher than the CV (Bishop et al., 2021; Exell et al., 2012).

The Shapiro-Wilk test confirmed the data's normality. The Pearson’s correlation was used to test the correlation between the metrics and the swimming performance at different distances. Bonferroni correction was applied to ensure that only significant results would be considered, with a new *p*-value of 0.016. The classification considered to interpret the correlation results was as follows: 0.00 to 0.30 negligible, 0.30 to 0.50 weak, 0.50 to 0.70 moderate, 0.70 to 0.90 strong, and > 0.90 very strong (Mukaka, 2012).

The World Aquatic points were calculated for each event (e.g., 50-, 100-, and 200-m front crawl) using the equation P = 1000 * (Base time / Swimmer time)^3^ (Santos et al., 2020).

## Results

Athletes' mean best swimming times were 25.2 ± 1.7 s for 50 m, 54.5 ± 3.4 s for 100 m, and 121.3 ± 8.4 s for 200 m, which corresponded to 522.7 ± 103.5, 570.5 ± 104.7, and 564.5 ± 110.7 World Aquatics points, respectively. Table 1 presents the descriptive data of the absolute side-to-side difference and relative asymmetry of the peak force, mean force, and impulse.

**Table 1:** Descriptive data of raw metric, absolute difference between sides and asymmetries.

Variables	Right	Left	Absolute ∆	Asymmetry (%)
Peak force (N)	226.4 ± 56.0	207.4 ± 53.9	33.7 ± 20.1	14.2 ± 8.0
Mean force (N)	143.1 ± 34.3	133.3 ± 34.0	17.3 ± 11.0	11.5 ± 6.7
Impulse (N.s)	40.2 ± 12.3	34.2 ± 10.7	9.0 ± 6.1	21.3 ± 13.8

Note: ∆ = difference

**Figure 1:**
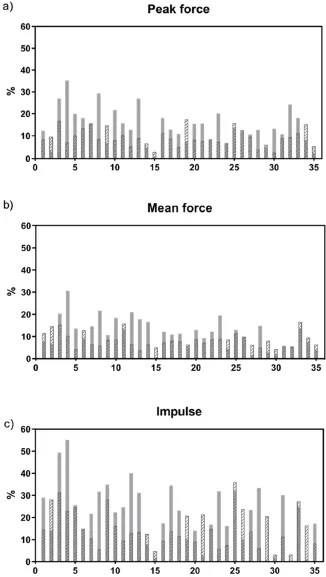
Individual asymmetries (solid grey bars) and coefficients of variations (hatched grey bars) for peak force (panel a), mean force (panel b), and impulse (panel c). Note: The asymmetry was considered “real” when a solid grey bar (relative asymmetry) had a higher value than a hatched grey bar (variable CV)

The individual relative asymmetries and CVs for the tested metrics are presented in Figure 1. The presence of “real” asymmetries was verified in 25 of the 35 (71.4%) athletes for peak force (panel a); 21 of the 35 (60%) athletes for mean force (panel b); and 22 of 35 (62.8%) athletes for impulse (panel c).

Table 2 presents correlations between peak force and swimming performance for the complete sample and only athletes with meaningful asymmetries. Peak force exhibited significant correlations with 50-m (strong—both sides), 100-m (moderate to strong—both sides), and 200-m (weak to moderate—both sides for the complete sample; left side for the athletes with “real” asymmetries) front-crawl. The peak force absolute side-to-side difference presented no significant correlation with swimming performance at any distance. Considering relative asymmetries, different results appeared for the correlations drawn with the complete sample and those drawn only with athletes with “real” asymmetries. When considering the whole sample, peak force relative asymmetries did not show significant correlations with swimming performance. However, when considering only athletes that showed meaningful asymmetries, the peak force asymmetry showed a significant positive correlation with 50-m (weak) and 100-m (moderate) front-crawl time (i.e., worst performance).

**Table 2:** Correlations (r) between peak force and swimming performance.

Swimming distance	Peak force(Right)	Peak force(Left)	Peak force absolute ∆	Peak force asymmetry
All athletes (n = 35)				
50 m	−0.78*	−0.77*	−0.26	0.04
100 m	−0.68*	−0.78*	−0.05	0.25
20 0m	−0.42*	−0.54*	−0.12	0.05
Only athletes with meaningful asymmetries (n = 25)				
50 m	−0.81*	−0.81*	0.01	0.47*
100 m	−0.71*	−0.83*	0.15	0.59*
200 m	−0.42	−0.58*	0.12	0.37

Note: ∆ = difference; * significant correlations (p ≤ 0.016)

Regarding the correlations between mean force and swimming performance (Table 3), significant correlations were seen between mean force and 50-m (strong—both sides), 100-m (moderate to strong—both sides), and 200-m (moderate—left side) front-crawl. Neither mean force absolute differences between sides nor mean force asymmetries showed significant correlations with swimming performance at any distance. The results were similar in both analyses, i.e., with the complete sample and with only athletes who presented real asymmetries.

**Table 3:** Correlations (r) between mean force and swimming performance.

Swimming distance	Mean force (Right)	Mean force (Left)	Mean force absolute ∆	Mean force asymmetry
All athletes (n = 35)				
50 m	−0.76*	−0.78*	−0.22	0.10
100 m	−0.69*	−0.79*	−0.07	0.22
200 m	−0.38	−0.51*	0.02	0.20
Only athletes with meaningful asymmetries (n = 21)				
50 m	−0.70*	−0.78*	−0.12	0.40
100 m	−0.67*	−0.84*	−0.08	0.38
200 m	−0.43	−0.60*	0.01	0.33

Note: ∆ = difference; * significant correlations (p ≤ 0.016)

The results for the correlations between impulse and swimming performance can be seen in Table 4. Moderate correlations between impulse and front-crawl swimming were only present at 50-m distance, when considering the complete sample or when considering only athletes with meaningful asymmetries. The exception was the “weak” correlation between impulse (left side) and 100-m front-crawl that was only exhibited when considering the complete sample. No significant correlations were seen between impulse absolute differences or impulse asymmetries and swimming performance.

**Table 4:** Correlations (r) between impulse and swimming performance.

Swimming distance	Impulse(Right)	Impulse (Left)	Impulseabsolute ∆	Impulseasymmetry
All athletes (n = 35)				
50 m	−0.56*	−0.50*	−0.32	−0.02
100 m	−0.39	−0.48*	−0.19	0.05
200 m	−0.21	−0.36	0.01	0.16
Only athletes with meaningful asymmetries (n = 22)				
50 m	−0.62*	−0.52*	−0.41	0.03
100 m	−0.43	−0.45	−0.24	0.14
200 m	−0.37	−0.35	−0.24	0.01

Note: ∆ = difference; * significant correlations (p ≤ 0.016)

## Discussion

Our study aimed to investigate the correlations between force, impulse, and their asymmetries (absolute and relative) with swimming performance across different distances, and to verify whether these relationships depended on the presence of “real” asymmetries. Our main findings were that: i) peak and mean force on both sides of the body were moderately to strongly associated with swimming performance at all distances (except 200 m—right side); ii) impulse was moderately correlated with 50-m swimming performance only; iii) percentage asymmetries of peak force correlated with swimming performance when considering only athletes with “real” asymmetries.

As expected, our results showed that stronger athletes (i.e., higher peak and mean force) presented faster swimming times in the tested distances. Our results were similar to those from other studies designed alike (dos Santos et al., 2017; Loturco et al., 2016; Morouço et al., 2015). According to Newton´s Laws of Motion, all movements are underpinned by force production. A swimmer’s successful locomotion in the water will largely depend on the interaction between the propelling limbs and the fluid environment (Santos et al., 2021). Therefore, if directed appropriately (i.e., in a technical manner and in a proper direction), more force applied in the water will typically result in more propulsion (Morais et al., 2022; Ruiz-Navarro et al., 2025), and likely, faster swimming times. Interestingly, it can be seen that the strength of the correlations gradually decreased from the 50- and 100-m distances to the 200-m distance. That suggests that as the event distance increased, other factors (e.g., aerobic capacity and pacing strategies) might play a more prominent role in the determinants of performance (Loturco et al, 2016). Corroborating that suggestion, Sokołowski et al. (2022) found that in a 200-m swimming test, the correlation between oxygen uptake (VO_2_) and the velocity in the 200-m distance (i.e., performance outcome) (r = 0.46 to 0.64) was higher than between the force in tethered swimming and the velocity in the 200 m (r = 0.32 to 0.34).

Impulse is the force applied during a certain amount of time, and it represents the change in momentum (momentum is responsible for changing the speed of a body). It was expected that correlations with swimming performance would be similar to those between force and performance. However, it was evident only for the shorter distance (i.e., 50 m: moderate correlation), suggesting that this metric correlates more with high-speed events. An analogous result was seen by Loturco et al. (2016) who highlighted that performance in a 50-m distance event demanded quick movements, which in turn were influenced by the ability of the muscles to contract fast. Thus, this evidences once again that in very short distances (i.e., 50 m), time-dependent variables (such as impulse) may have a greater influence on the performance in comparison to longer distances (i.e., 100 and 200 m). Another factor that can help in understanding the results is that impulse is a metric with more variability than force, as can be seen by SD values, which in turn can flatter the slope of the distribution of swimmers, blunting the magnitude of the correlations. These results somehow reinforce that force is likely a more robust metric, exhibiting less variability, and should be considered during swimming performance testing and monitoring.

In general, when considering the whole sample, no significant correlations were seen between force asymmetries (absolute or relative) measured during tethered swimming and swimming performance (i.e., times in 50, 100 and 200 m). In addition, significant correlations were also not seen between impulse asymmetries and swimming performance (for both, the whole sample and only athletes with “real” asymmetries). These findings are in accordance with other studies on the topic, in which relative asymmetries did not exhibit associations with performance in swimming (Morouço et al., 2015; Psycharakis et al., 2021). An interesting example is the study by Santos et al. (2024), which measured asymmetries in in-water hand force over an entire season. The asymmetries were shown to increase during the season (from 17.1 to 28.4%), yet there were no significant correlations between asymmetry values and 25-m velocity at any point in the season. A few reasons are suggested to help explain the results. This lack of significant correlations may be due to the natural variability of asymmetries, as shown by the large SD relative to the mean (Bishop et al., 2021). Thus, for example, when plotting data from a stable metric (e.g., swimming time) against a highly variable metric (e.g., asymmetry), the slope is rarely going to present a strong association. Another reason may be that the between-limb imbalances in these metrics are not as important as the capacity itself, the total output being more relevant than the between-limbs asymmetries. In this sense, other aspects, such as physiological and biomechanical factors, and training history, may perhaps be more prominently correlated to performance than asymmetries. Lastly, Psycharakis et al. (2021) suggested that the interlimb asymmetries of force production in swimming may be a compensation for asymmetries elsewhere in an attempt to maintain performance. Thus, the overall performance would not be affected by the presence of interlimb differences. Specifically regarding impulse, when considering that even the raw data did not present an association with performance (100- and 200-m front-crawl), it is not surprising that the asymmetries also did not show a significant relationship, since ratio data almost always exhibit notably greater noise and measurement error (Bishop, 2025).

Nevertheless, an interesting finding of the present study was that significant correlations appeared between peak force relative asymmetries and swimming performance at 50-m (weak) and 100-m (moderate) front-crawl swimming when analysing only athletes presenting “real” asymmetries. These findings help to support that when the metric becomes more stable (i.e. less variability means less chance of data error), the correlations can be more clearly explored as the results are more robust. Based on the findings, it may be suggested that, at short distances, similar peak force applications between the body sides might be relevant for improved performance, especially in the 100-m distance, where the strength of the correlation was higher (moderate). It is interesting to notice that these associations were not seen at the 200-m distance, once again suggesting that as the event distance increases, other aspects (such as raw metrics and/or physiological factors) may be more relevant for performance. Although the peak force asymmetry was the only metric significantly associated with performance when using this approach, the magnitude of the correlations with mean force increased substantially when compared with the analysis using the complete sample. Thus, it seems that this approach could be more explored in future research, as the correlations between asymmetries and performance may present different results depending on the presence of “real” asymmetries (or not).

Although important data with new approaches are brought, the present manuscript is not without limitations. Firstly, even though the official swimming performance in 50-, 100- and 200-m front crawl was obtained from the National Aquatic Confederation records, the time between the data collection and the event performance could have been up to a year in some instances, which might have an impact on the results and should be considered when interpreting our findings. In addition, the sample included non-professional youth and adult athletes, which may have skewed the data. Finally, the use of tethered swimming may lead to different stroke mechanics than in “free” swimming. Although that is a validated and reliable method, this should be considered in the interpretation of the results. Future research is needed to fill some remaining gaps about the topic. For example, a controlled study with an experimental and longitudinal design is needed to explore whether a training intervention to reduce peak force asymmetries is relevant for performance. Also, only short to middle distances were investigated as a measure of performance in the present study, and longer distances, such as 400, 800, and/or 1500 m, could provide distinct results and are encouraged for future studies. Future research should also seek to investigate the topic in female athletes and to explore whether side-to-side differences may play a role in the appearance of injuries in swimmers.

## Conclusions

In summary, the raw metrics of force and impulse on both sides presented negative strong to moderate correlations with swimming performance, showing that, in general, stronger athletes have faster swimming times. Absolute side-to-side differences and relative asymmetries did not present associations with swimming performance when considering the whole sample, but when considering only athletes presenting “real” asymmetries, significant weak to moderate relations were seen with short-distance swimming performance. These findings highlight the need for individual analysis of the asymmetries, as well as the consideration of the metric variability.
